# The Need to Identify Novel Markers for Early Renal Injury in Cardiorenal Syndrome

**DOI:** 10.3390/cells13151283

**Published:** 2024-07-30

**Authors:** Anna Lisa, Federico Carbone, Luca Liberale, Fabrizio Montecucco

**Affiliations:** 1 First Clinic of Internal Medicine, Department of Internal Medicine, University of Genoa, 6 Viale Benedetto XV, 16132 Genoa, Italyfederico.carbone@unige.it (F.C.); luca.liberale@unige.it (L.L.); 2 IRCCS Ospedale Policlinico San Martino, Genoa-Italian Cardiovascular Network, 10 Largo Benzi, 16132 Genoa, Italy

**Keywords:** cardiorenal syndrome, acute kidney injury, chronic kidney disease, heart failure, biomarkers

## Abstract

The term “Cardiorenal Syndrome” (CRS) refers to the complex interplay between heart and kidney dysfunction. First described by Robert Bright in 1836, CRS was brought to its modern view by Ronco et al. in 2008, who defined it as one organ’s primary dysfunction leading to secondary dysfunction in the other, a view that led to the distinction of five different types depending on the organ of primary dysfunction and the temporal pattern (acute vs. chronic). Their pathophysiology is intricate, involving various hemodynamic, neurohormonal, and inflammatory processes that result in damage to both organs. While traditional biomarkers have been utilized for diagnosing and prognosticating CRS, they are inadequate for the early detection of acute renal damage. Hence, there is a pressing need to discover new biomarkers to enhance clinical outcomes and treatment approaches.

## 1. Introduction

The term “Cardiorenal Syndrome” (CRS) encompasses a complex interplay of dysfunction within both the cardiac and renal systems, presenting with variable temporal patterns depending on the specific subtype of the syndrome. Dysfunction may manifest acutely, chronically, or as a component of a systemic disease affecting both organs. This intricate relationship was first hypothesized by Robert Bright in 1836, where he observed structural cardiac changes in patients with advanced renal insufficiency. In 1840, a case series highlighted the correlation between cardiovascular pathologies and renal impairments, characterized by heightened albumin secretion [1]. In 2004, the National Heart, Lung, and Blood Institute (NHLBI) defined CRS as an outcome of intricate interactions between the renal and cardiac systems, culminating in the increased circulatory volume and exacerbation of heart failure symptoms alongside disease progression.

**Figure 1:**
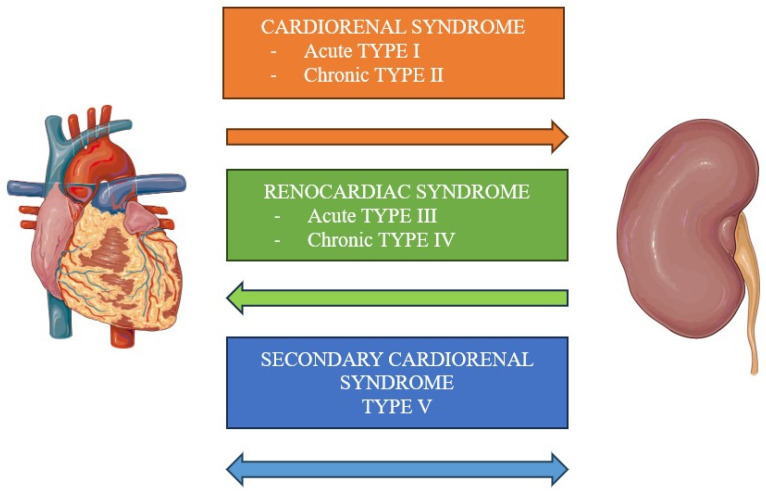
Classification of cardiorenal syndromes. CRSs are classified based on the primary involvement of the kidney or the heart and based on its temporal progression. Type I and II include cardiac conditions causing a secondary damage to the kidney in an acute or chronic fashion, respectively. Type III and IV encompass kidney dysfunction that damages the heart in an acute or chronic fashion, respectively. Finally, type V includes systemic afflictions that affects both cardiac and kidney functions.

In 2008, Ronco et al. provided a formal definition of CRS as primary dysfunction in one organ, occurring either acutely or chronically, leading to secondary dysfunction in the other one [2]. While CRS underscores the intimate physio-pathological interconnection between the heart and kidneys, the Acute Dialysis Quality initiative proposed a refined classification system, dichotomizing the syndrome into distinct groups based on the origination of primary organ dysfunction that triggers the pathological cascade (cardiorenal or renocardiac) [3]. These subgroups are further subdivided based on the acuity or chronicity of organ involvement, except for a category implicating systemic causes (Figure 1).

Unravelling the causal nexus between primary and secondary organ dysfunction can prove challenging, particularly in the presence of concurrent risk factors such as diabetes, hypertension, and atherosclerosis that intricately influence both systems, thereby shaping a unified clinical profile [4]. The bidirectional interplay of cardiac and renal dysfunction initiates a cascade of feedback mechanisms culminating in detrimental outcomes for both organ systems. Pathogenesis involves a complex interplay of hemodynamic, neurohormonal, and inflammatory mediators predominantly culminating in volume overload [5]. Although data are scarce, CRS can associate with high mortality rates depending on the underlying cause and the type. In a retrospective cohort study, acute CRS was associated with a higher risk of death compared with chronic renocardiac syndrome or chronic kidney disease (CKD) without CRS [6]. Yet, existing diagnostic criteria are deemed insufficient in this regard and urgent attention is warranted towards the discovery and validation of novel, highly predictive biomarkers to facilitate accurate assessment, monitoring, and tailored therapeutic strategies for CRS [7].

### 1.1. Renal Diagnostic Criteria

In the context of heart failure, volume overload often leads to alterations in serum creatinine levels, which may sometimes be within normal range or even decreased. Consequently, this biomarker is incorporated into the diagnostic criteria established by the Kidney Disease Improving Global Outcomes (KDIGOs) for both acute kidney injury (AKI) and CKD. However, there exists a distinct clinical entity presenting with acute kidney disturbances that do not meet the criteria for either AKI or CKD, termed acute kidney disease (AKD). These presentations vary based on temporal patterns. AKD is characterized by structural and functional abnormalities lasting ≤3 months. Conversely, AKI is considered a subgroup of AKD, defined as a renal function abnormality over a period of 6 h to 1 week. CKD is understood as the presence of both structural and functional renal abnormalities with systemic involvement lasting ≥3 months. Functional criteria for AKI include: a >50% increase in serum creatinine over the past 7 days, or a ≥0.3 mg/dL increase in serum creatinine within 2 days, or oliguria lasting ≥4 h. AKD presents the same functional criteria as AKI picture but lists also glomerular filtration rate (GFR) <60 mL/min/1.73 m^2^, or a ≥35% reduction in GFR compared to baseline, or a ≥ 50% increase in serum creatinine compared to baseline. In this case, structural criteria include markers of renal damage such as albuminuria, acid–base and electrolyte disturbances, haematuria, or sediment abnormalities. Lastly, such structural and functional abnormalities are also shared in CKD, which is differentiated based on the temporal pattern as those last ≥3 months [8] (Table 1).

### 1.2. Cardiac Diagnostic Criteria

Heart failure (HF) is a clinical syndrome characterized by symptoms and signs resulting from a structural and/or functional cardiac abnormality, stemming from an alteration in systolic and/or diastolic function leading to reduced cardiac output and/or increased intracardiac pressures at rest or during stress. The most commonly used classification is based on the measurement of left ventricular ejection fraction (LVEF) and describes patients with LVEF ≥50% (heart failure with preserved EF, HFpEF), LVEF < 40% (heart failure with reduced EF, HFrEF), and LVEF between 40 and 49% (heart failure with mid-range ejection fraction, HFmrEF) [9]. Furthermore, based on the temporal course, heart failure can be differentiated into de novo and acute versus chronic.

## 2. Classification

The Acute Dialysis Quality Initiative group proposed a classification of CRS, dividing it into two groups based on which organ was primarily involved: heart and then kidney, or kidney and then heart. Each type was further subdivided based on the temporal course: acute form (type 1 and 3) and chronic form (type 2 and 4). Finally, type 5 encompasses the condition of systemic damage involving both the kidney and the heart [10,11] (Figure 1). This classification can be very useful in setting up the therapeutic pathway, but it is important to keep in mind that there is often overlap between the different groups, with the evolution from one type to another during the progression of the disease.

### 2.1. Type I

**Figure 2:**
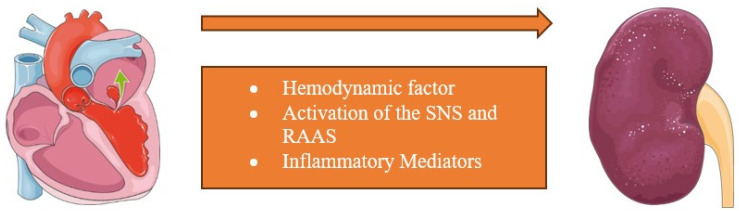
Underlying mechanisms for CRS type I and type II. CRS type I and II are due to cardiac afflictions primarily altering kidney function. Its pathogenesis involves the increased activation of the sympathetic nervous system (SNS), as well as a rise in inflammatory mediators and the renin–angiotensin–aldosterone system (RAAS). Additionally, hemodynamic alterations such as renal hypoperfusion play a significant role.

The CRS type I, or acute cardiorenal syndrome, is characterized by the development of AKI in patients with acute cardiac diseases; this is, in most cases, acute decompensated heart failure (ADHF). The most common causes include acute coronary syndrome, pulmonary embolism, pericardial tamponade, myocarditis, papillary muscle rupture, and arrhythmias. These conditions result in acute heart failure characterized by hemodynamic alterations such as renal hypoperfusion, which plays a significant role in this category [12]. Consequently, there is a persistent activation of the sympathetic tone, as well as a rise in inflammatory mediators and the renin–angiotensin–aldosterone system (RAAS), which may worsen both cardiac and renal function [13] (Figure 2).

### 2.2. Type II

In CRS type II, or chronic cardiorenal syndrome, chronic HF is responsible for the onset or progression of CKD. Indeed, CKD is observed in 45% to 63% of patients with chronic heart failure and may represent the evolution of CRS type I [14,15,16]. In this category as well, the systemic and local activation of the SNS and RAAS, hemodynamic factors of renal hypoperfusion, chronic low-grade inflammation, and venous congestion appear to play a predominant role [17,18] (Figure 2). 

### 2.3. Type III

**Figure 3:**
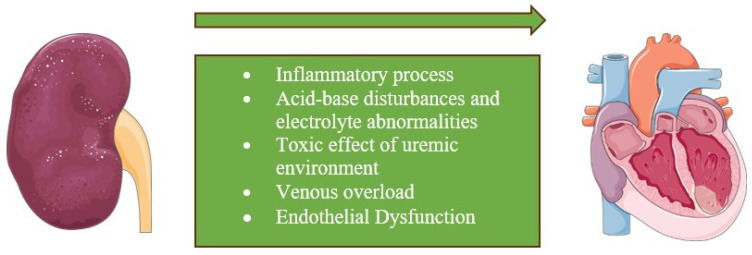
Underlying mechanisms for the CRS type III and type IV. CRS type III and IV are characterized by acute or chronic alterations in renal function leading to cardiac disease. The underlying pathophysiology is not well understood but suggests a bidirectional relationship between the kidney and heart, involving inflammatory processes and physiological imbalances such as acid–base disturbances, electrolyte abnormalities, volume overload, chronic inflammation, endothelial dysfunction, and the toxic effects of the uremic environment.

CRS type III, or acute renocardiac syndrome, is characterized by an acute alteration in renal function leading to cardiac dysfunction [10], including ADHF, acute myocardial infarction (AMI), and cardiac arrhythmias [19]. The underlying pathophysiology is not well understood. Existing evidence suggests a bidirectional relationship between these two systems, involving both the direct effects of AKI on the heart and the effects of AKI on the function of other organs with indirect effects on the heart. Such mechanism involves triggering an inflammatory process leading to cytokine activation, leukocyte infiltration, and apoptotic cell death, resulting in compromised cardiac function. Additionally, known physiological imbalances such as acid–base disturbances, electrolyte abnormalities, and volume overload are associated with this condition [20] (Figure 3).

### 2.4. Type IV

CRS type IV, also known as chronic renocardiac syndrome, represents a category characterized by progressive alterations in renal function leading to cardiac diseases. The association between CKD and increased CV risk has long been recognized. Studies indicate that cardiovascular causes account for nearly 50% of deaths across all age groups of patients with CKD [21]. The pathogenesis involves a complex interaction of factors common to both CKD and cardiovascular disease. These include modifiable risk factors such as cigarette smoking, dyslipidemia, age, and diabetes [22]. Other factors are involved in the process of chronic inflammation responsible for endothelial dysfunction and/or the toxic effect of the uremic environment [11] (Figure 3). 

### 2.5. Type V

**Figure 4:**
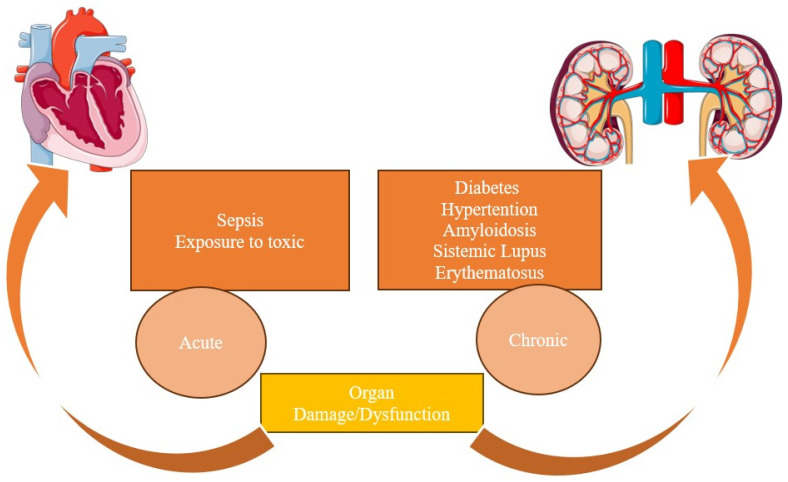
Underlying mechanisms for CRS type V. CRS type V occurs due to simultaneous kidney and heart dysfunction in systemic conditions. It can be acute, often due to sepsis or toxic drugs, or chronic, commonly associated with diabetes, hypertension, or amyloidosis.

CRS type V is secondary to the simultaneous involvement of both the kidney and the heart in systemic clinical conditions. CRS can occur both acutely and chronically without primary or secondary organ dysfunction. In acute forms, the most frequent conditions are sepsis, infections, or exposure to toxic drugs. Meanwhile, chronic conditions may include diabetes mellitus, hypertension, and systemic amyloidosis, for example. However, some conditions such as systemic lupus erythematosus can present acutely and chronically. Moreover, diabetes mellitus can involve either the heart or the kidney at different times, making it challenging to categorize into a specific CRS category [10] (Figure 4).

## 3. Pathophysiology

The pathophysiology of CRS is characterized by a series of processes responsible for damage at both the cardiac and renal levels. The list of contributors to CRS onset and progression include common cardiac and renal risk factors such as hypertension, diabetes, atherosclerosis, chronic inflammation, obesity, dyslipidemia, older age, sex, and smoking [23]. The underlying mechanisms involve a cascade of events that affect both the heart and the kidneys, leading to a mutual amplification of damage. These processes involve the interaction of multiple factors, both hemodynamic and non-hemodynamic, which are not always fully understood.

### 3.1. Hemodynamic Alterations

#### 3.1.1. Venous Congestion

Many pieces of evidence demonstrate how the role of renal venous congestion is a primary factor in CRS. The earliest studies date back to 1913 with Rowntree et al. [24] and then Winton [25]. Later, in 1949, Blake [26] and colleagues documented how the increase in renal venous hypertension alters renal hemodynamic parameters such as plasma flow and glomerular filtration rate, leading to significant reductions in sodium excretion. A post hoc analysis of the ESCAPE (Evaluation Study of Congestive Heart Failure and Pulmonary Artery Catheterization Effectiveness) demonstrated that right atrial pressure was the only hemodynamic parameter associated with renal dysfunction [27]. The increase in central venous pressure (CVP) caused by ADHF results in renal venous congestion, reducing blood flow through renal vasculature. The mechanisms responsible for worsening renal function and venous congestion include increased renal interstitial pressure, tubular obstruction, and the activation of the renin–angiotensin system and sympathetic nervous system, resulting in increased sodium avidity [28]. 

#### 3.1.2. Intra-Abdominal Pressure

Elevated intra-abdominal pressure, assessed using a bladder catheter connected to a transducer, correlates with deteriorating kidney function in acute heart failure patients [29]. The compressive impact on renal veins and ureters, resulting in decreased renal filtration, may elucidate the contribution of elevated intra-abdominal pressure to the pathogenesis of CRS [29,30].

### 3.2. Non-Hemodynamic Alterations

#### 3.2.1. Neurohormonal Pathways

Among the mechanisms contributing significantly to cardiac or renal dysfunction in CRS is the activation of the sympathetic nervous system (SNC) and the RAAS. The decrease in arterial circulating blood volume triggers neurohormonal activation, including RAAS, the endothelin system, and arginine vasopressin. These systems induce water retention through sodium-retentive vasoconstriction, countered by vasodilatory natriuretic hormone systems and cytokines [31,32]. Normally, these mechanisms work together to maintain vascular tone, cardiac output, and tissue perfusion. However, in heart failure, they perpetuate vicious cycles leading to chronic renal hypoxia, inflammation, and oxidative stress, which can independently alter cardiac and renal structure and function [33].

#### 3.2.2. Oxidative Stress

Under normal circumstances, ROS are produced in a balanced manner in all organs, including the heart and kidneys, for cellular functions [34,35]. However, during pathological or physiological stress, disruptions in oxidative reaction homeostasis can lead to increased ROS production by mitochondria, causing tissue damage [36]. Impaired mitochondrial metabolism in cardiomyocytes and kidney tubular cells represents the ultimate convergent pathway leading to tissue injury in CRS patients [37].

#### 3.2.3. Inflammation

Inflammatory processes may be implicated in the pathogenesis of CRS, contributing to cellular damage in both the heart and kidneys. Both chronic kidney disease and heart failure entail pronounced chronic inflammation, leading to the generation of pro-inflammatory biomarkers, which play a crucial role in tissue damage to both organs, resulting in cellular death and fibrosis. Key triggers initiating and propagating the inflammatory cascade include the activation of the sympathetic nervous system and the RAAS, venous congestion, ischemia, and oxidative stress [5]. 

Specifically, the circulating inflammatory mediators shown to rise after AKI, such as TNF-α (tumor necrosis factor-α), IL-1 (interleukin-1), and IL-6 (interleukin-6), have direct depressant effects on the heart and have been shown to reduce left ventricular ejection fraction and cause long-term ventricular remodeling [38]. Furthermore, following the rise of inflammatory mediators and with the help of stretch stress due to peripheral venous congestion, the endothelium is dysfunctionally activated switching toward a proinflammatory and prothrombotic phenotype [39]. Recently, a role for kidney dendritic cells and their crosstalk with cardiac homologous has emerged with an important role in CRS [40]. Independently of the underlying cause, chronic renal damage is characterized by some degree of renal cell necrosis with inflammatory response [41]. Different mechanisms of necrosis have been involved in the progression of kidney damage including ferroptosis and necroptosis, all leading to an inflammatory response characterized by the local immune system and endothelial cell activation with a rise in local oxidative stress and the release of pro-inflammatory cytokines [42]. Indeed, after the injury, the remaining cells are dedifferentiated and proliferate to replace the damaged tissue. Yet, under the conditions of chronic damage, the kidneys cannot generate new nephrons, and maladaptive response leads to further injury [43]. Indeed, maladaptive repair leads to failed tubule recovery, a decrease in epithelial cells and an increase in the mesenchymal ones, enhancing fibrosis and resulting in CKD. Myofibroblast and damaged tubular cells produce pro-fibrotic factors such as TGF-β and fibroblast growth factor-23 (FGF-23), facilitating the formation of fibrotic scars [44]. Fibrosis, in turn, associates with capillary rarefaction and local hypoxia, resulting in further damage. FGF-23 recently showed an important role in mediating cardiac hypertrophy following renal injury [45]. After hypertrophy onset, microvascular ischemia may also participate in the progression of cardiac damage while profibrotic factors released at the renal site may reach the heart and precipitate similar pathways at distance [46].

Of much interest, oxidative stress and inflammation contribute to the onset of endothelial dysfunction by damaging the endothelial cells lining blood vessels, leading to impaired vasodilation, increased permeability, and promoting thrombosis and atherosclerosis [47]. Endothelial dysfunction is, therefore, a critical factor in the development and progression of CRS by altering blood flow and modifying the physiological responses of neurohormonal axis, which further heightens inflammatory responses and oxidative stress, therefore, facilitating the onset of a vicious circle [48].

## 4. Biomarkers of Renal Injury

Biomarkers have traditionally been used in the diagnostic and prognostic process of CRS. However, the currently available biomarkers do not always prove effective in identifying early acute renal damage associated with heart diseases [49], potentially slowing down the diagnostic process. Moreover, the same biomarkers of renal or cardiac damage present several limitations in projecting the progression of CRS in a chronic context. 

### 4.1. Established Biomarkers

#### 4.1.1. Creatinine

One of the standard parameters used for the diagnosis of AKI is serum creatinine; yet, this is not reliable during acute changes of renal function [50]. Serum creatinine (sCr) would be more of a marker of function rather than injury, and its concentration does not increase until there is a moderate loss of renal function. Additionally, sCr is influenced by muscle mass. Therefore, in the elderly, malnourished, or chronically ill patients, it may remain within range even in the presence of AKI. Furthermore, in cases of volume overload such as in ADHF, sCr may be falsely low, delaying the diagnostic phase. In CRS, there is tubular injury with cell death before renal function loss occurs [49]. Hence, there is a need to identify new renal markers that indicate tubular injury earlier than creatinine [51].

#### 4.1.2. Glomerular Filtration Rate (GFR)

Glomerular filtration rate (GFR) serves as an index of renal function, measured as the rate of plasma filtration in the nephron over a certain period of time [52]. Its estimate (eGFR) is part of the diagnostic criteria of CKD. Reduction in eGFR in CRS patients correlates with negative outcomes and increased mortality [53]. However, during volume reduction in patients undergoing diuretic therapy for ADHF, eGFR may not be that accurate. Additionally, eGFR is calculated considering sCR, which, as previously discussed, can be misleading, thus delaying diagnosis [52].

#### 4.1.3. Brain Natriuretic Peptide

The measurement of B-type natriuretic peptide (BNP) in the blood involves both its forms, BNP, which is biologically active, and N-terminal (NT) pro-hormone BNP (NT-proBNP), the inactive precursor. BNP is a peptide synthesized and released by ventricular cardiomyocytes, with functions including diuresis and natriuresis. BNP and NT-proBNP serve as diagnostic biomarkers in ADHF and prognostic markers in chronic heart failure. Studies conducted by Takahama et al. [54] have shown that the increase in the NT-proBNP/BNP ratio precedes the worsening of renal function in patients with ADHF and is associated with a decrease in GFR. The NT-proBNP increases with age and reflects age-related health status. In older individuals without cardiac issues, the BNP/NT-proBNP level can be significantly higher than baseline levels [55], thereby reducing its utility as a sole biomarker for CRS diagnosis.

### 4.2. Novel Biomarkers (Table 2)

#### 4.2.1. Neutrophil Gelatinase-Associated Lipocalin (NGAL)

NGAL is synthesized and expressed by proximal and distal renal tubular cells [56] following stress conditions, particularly in response to infections, inflammation, ischemia, or neoplastic transformation [57,58,59,60]. NGAL is also secreted at basal levels by cardiomyocytes and other tissue cells [58]. Being produced within the kidney during ischemic conditions, NGAL is seen as a potential marker of acute renal tubular injury and necrosis [61]. Moreover, NGAL can be detected both in blood and urine [61,62]. From the currently available studies, it has been demonstrated that an increase in the biomarker in urine and blood has allowed for the detection of AKI in patients with ADHF well before any significant changes in sCr levels [63].

#### 4.2.2. Cystatin C (CysC)

CysC is a cysteine protease inhibitor protein released into the bloodstream by all nucleated cells [64]. CysC is filtered through the glomerulus into the urine and subsequently completely reabsorbed at the proximal tubule level without being secreted, making it a parameter of renal function. Its values are not influenced by age, sex, or muscle mass, unlike creatinine [65,66]. Shardlow et al. have shown that CysC is a better marker of renal function in chronic kidney disease compared to creatinine [67]. Furthermore, in acute heart failure and CRS scenarios, increased levels of cystatin C prove to be a reliable indicator for predicting outcomes after discharge, offering a valuable means of categorizing patients according to their risk of adverse post-discharge events [68].

#### 4.2.3. Kidney Injury Molecule-1 (KIM-1) 

KIM-1 is a type 1 transmembrane receptor glycoprotein that is expressed following tubular damage on the surface of proximal tubular epithelial cells, while under physiological conditions, it is not expressed. It can be measured in urine [69,70]. There are studies showing that in AKI, the increase in urinary KIM-1 can be used as a potential biomarker for the acute diagnosis of CRS [71]. Specifically, KIM-1 can be used as an additional biomarker alongside other markers in AKI, especially in its ischemic or nephrotoxic form, enhancing their sensitivity and specificity [58,59,60].

#### 4.2.4. N-Acetyl-β-d-Glucosaminidase (NAG)

NAG is a lysosomal enzyme located on the brush border of the epithelial cells of the proximal tubule, which under normal conditions is not detected in urine. It degrades glycoproteins and glycosaminoglycans, facilitating the breakdown of complex carbohydrates in lysosomes [72]. It is not released by cells, and, due to its high molecular weight, it is not filtered by the glomerulus [73]. Consequently, its urinary levels specifically reflect the extent of tubular damage in the event of nephron injury during AKI [74,75,76].

#### 4.2.5. Interleukin 18 (IL-18)

IL-18 is a pro-inflammatory cytokine that enhances the activity of natural killer cells and T-cells, playing a crucial role in the immune response against infections and tumors. Additionally, IL-18 promotes the production of other cytokines, such as interferon-gamma (IFN-γ), and is involved in the pathogenesis of inflammatory diseases by contributing to the inflammatory cascade. IL-18 is early released in urine during the acute ischemic damage of the proximal renal tubules, and its levels rise 48 h before serum creatinine levels [77,78].

#### 4.2.6. Galectin-3

Galectin-3 (Gal-3) belongs to the family of beta-galactosidase-binding lectins [79]. It is released by macrophages, stimulating collagen activation and deposition in the extracellular matrix [80,81]. Therefore, its main role is to promote fibrosis; particularly in the cardiac context, it can lead to the remodeling and progression of heart failure. Moreover, Gal-3 is also involved in renal fibrosis and dysfunction, where an increase in serum levels may precede the reduction in GFR [82]. Similarly, in patients with chronic heart failure, elevated serum levels of Gal-3 are associated with an increased risk of worsening renal function [83].

**Table 2:** List of biomarkers linked to CRS outcomes and their characteristics.

Novel Biomarker	Description	References	Results
**NGAL**	Marker secreted in urine and blood. An early marker of renal damage.	Song et al. [84]	Diagnosis of CRS type I: ROC curve AUC 0.875 [0.813–0.937] *p* < 0.001
		Alvelos et al. [85]	Development of CRS type I in patients with acute heart failure: AUC 0.93 [0.88–0.98] *p* < 0.001
		Chen et al. [86]	AKI progression in patients with CRS type I: OR, 4.7; 95% CI, 1.7–13.4 *p* < 0.001
**CysC**	Assessment of kidney function. In CKD, it is better than serum creatinine. Predictor of adverse outcomes	Pinsino et al. [87]	CysC-based estimated glomerular filtration rate predicts a composite endpoint of in-hospital mortality, renal replacement therapy, or severe right ventricular failure in patients with LVAD: OR per 5 mL/(min·1.73 m^2^) decrease 1.16 (1.02–1.31)
		Ruan et al. [88]	Levels of Cys C are independently associated with in-hospital and 12-months mortality in patients with CRS type I OR, 1.48; 95% CI, 1.75–4.16, *p* = 0.027 and OR, 2.72; 95% CI, 1.92–4.28, *p* = 0.017, respectively
		Rafouli-Stergiou et al. [68]	In-hospital changes in CysC predicted cardiac death or rehospitalization for heart failure decompensation at 60 days in patients with CRS: ROC curve, AUC 0.681 [0.549–0.812], *p* = 0.014
**NAG**	Marker of acute kidney injury.	Liangos et al. [74]	The second, third, and fourth quartile groups of NAG associated with an increased risk of dialysis requirement or hospital death in patients with AKI: OR 3.0 (95% CI 1.3–7.2); OR 3.7 (95% CI 1.6–8.8) and OR 9.1 (95% CI 3.7–22.7), respectively
**KIM-1**	Marker of acute kidney injury. It is measured in urine.	Liangos et al. [74]	The second, third, and fourth quartile groups of KIM associated with an increased risk of dialysis requirement or hospital death in patients with AKI: OR 1.4 (95% CI 0.6–3.0), OR 1.4 (95% CI 0.6–3.0), and OR 3.2 (95% CI 1.4 to 7.4), respectively
		Kaddourah et al. [71]	In children with dilated cardiomyopathy, a combined model using cut-off values of KIM-1 ≥ 235, IL-18 ≥ 17.5, and (BNP) > 15 pg/mL resulted in a distinction between patients with mildly depressed LV (55 > LVEF ≥ 45) and those with LVEF < 45%: ROC curve AUC 0.70
**IL-18**	Early marker of acute kidney injury.	Parikh et al. [77,78]	Levels of IL-18 predicted the development of AKI in ICU patients: IL-18 > 100 pg/mL OR 6.5 (95% CI 2.1–20.4) *p* < 0.001 Levels of IL-18 predicted mortality in ICU patients: IL-18 >200 pg/mL OR 2.32 (95% CI 1.2–4.4) *p* < 0.001
		Chen et al. [86]	AKI progression in patients with CRS type I: OR 3.6 (95% CI 1.4–9.5)
		Kaddourah et al. [71]	In children with dilated cardiomyopathy, a combined model using cut-off values of KIM-1 ≥ 235, IL-18 ≥ 17.5, and (BNP) > 15 pg/mL distinguished patients with mildly depressed LV (55 > LVEF ≥ 45) and those with LVEF < 45%: ROC curve AUC 0.70
**Gal-3**	Marker of cardio-renal fibrosis and dysfunction.	Iacoviello et al. [83]	Gal-3 associated with kidney injury in patients with chronic heart failure: OR 1.08 (95% CI 1.02–1.14), *p* = 0.012

Abbreviations: AKI, acute kidney injury. LV, left ventricle. LVEF, left ventricle ejection fraction. NGAL, neutrophil gelatinase-associated lipocalin. NAG, urinary enzyme N-acetyl-β-D-glucosaminidase. KIM-1, kidney injury molecule-1. IL-18, interleukin 18. Gal-3, galectin-3. OR, odds ratio. 95% CI, 95% confidence of interval. AUC, area under the curve.

## 5. Conclusions and Perspectives

CRS is a complex condition classified into five types based on the heart–kidney relationship and is associated with high morbidity and mortality, particularly when undiagnosed early. While numerous studies have explored potential pathophysiological mechanisms involving both organs, many aspects remain unclear. Traditional diagnostic criteria often rely on biomarkers to detect renal insufficiency or heart failure, whether acute or chronic, highlighting the need for effective biomarkers that can diagnose cardiac dysfunction in renal diseases and renal damage in heart failure.

The current biomarkers for CRS are limited due to a lack of significant studies, complicating their application across different CRS types. A promising strategy involves using multiple biomarkers to improve diagnostic accuracy, though this requires further validation.

This review aims to identify new biomarkers that could aid in the diagnostic, therapeutic, and prognostic processes. Traditional renal markers like creatinine, GFR, and BNP/NT-proBNP are evaluated, with their limitations noted. New biomarkers such as NGAL, CysC, KIM-1, NAG, IL-18, and Gal3 show potential in identifying acute kidney injury and some as prognostic markers. Ongoing research aims to better assess renal and cardiac dysfunction, with future studies expected to validate these new biomarkers as diagnostic and therapeutic targets.

## Figures and Tables

**Table 1:** Renal damage definition and criteria by the Kidney Disease Improving Global Outcomes (KDIGOs).

	AKI	AKD	CKD
**Temporal Pattern**	≤7 days	≤3 months	≥3 months
**Functional Criteria**	Increase in serum creatinine ≥ 50% within 7 days, or increase ≥0.3 mg/dL within 2 days, or oliguria for ≥4 h.	The same as AKI, or GFR < 60 mL/min/1.73 m^2^, or decrease in GFR by ≥35% with respect to baseline, or increase in serum creatinine by ≥50% with respect to baseline.	GFR < 60 mL/min/1.73 m^2^
**Structural Criteria**		Albuminuria, hematuria, acid–base and electrolyte disturbances or sediment abnormalities.	

Abbreviations: AKD: acute kidney disease, AKI: acute kidney injury, CKD: chronic kidney disease, GFR: glomerular filtration rate.
